# Antimicrobial Susceptibility and Virulence Surveillance of *Campylobacter* spp. Isolated From Patients in Two Tertiary Medical Centers in Taiwan

**DOI:** 10.3389/fmicb.2018.03186

**Published:** 2019-01-07

**Authors:** Mao-Cheng Ge, Shu-Fang Kuo, Shih-Cheng Chang, Chun-Chih Chien, Huey-Ling You, Jang-Jih Lu

**Affiliations:** ^1^Department of Laboratory Medicine, Chang Gung Memorial Hospital, Taoyuan, Taiwan; ^2^Department of Medical Biotechnology and Laboratory Sciences, College of Medicine, Chang Gung University, Taoyuan, Taiwan; ^3^Department of Laboratory Medicine, Kaohsiung Chang Gung Memorial Hospital, Kaohsiung, Taiwan; ^4^Department of Medicine, College of Medicine, Chang Gung University, Taoyuan, Taiwan

**Keywords:** *Campylobacter*, antibiotic susceptibility, virulence factor, disk diffusion, *E*-test

## Abstract

*Campylobacter* spp. may cause fever, vomiting, and diarrhea in humans. Antibiotic treatment is suggested for patients with severe campylobacteriosis. However, the interpretative criteria for antibiotic susceptibility are inconsistent between Clinical and Laboratory Standards Institute and European Committee on Antimicrobial Susceptibility Testing guidelines. The aim of the study is to investigate the antibiotic susceptibility and prevalence of cytolethal distending toxin genes and to evaluate antibiotic susceptibility testing methods in the clinical laboratories of two tertiary medical centers in Taiwan. In total, 236 bacterial isolates were collected between 2001 and 2014. The disk diffusion and *E*-test methods were used to evaluate the antibiotic susceptibility, and broth dilution results were used as a reference. The virulence genes *cdtA, cdtB, cdtC*, and *ceuE* were detected through polymerase chain reaction. The antimicrobial sensitivity rates for erythromycin, ciprofloxacin, and tetracycline using the broth dilution assay were 80.4, 5.4, and 3.4%, respectively. No significant differences were observed in the antibiotic susceptibility of the isolates obtained from southern and northern Taiwan. However, some differences were observed between species. The susceptibility test for erythromycin (disk diffusion) showed that the isolates with small inhibition zone diameters were all resistant, and five isolates (4.0%) with large IZDs were non-sensitive. The error rate of the disk diffusion method according to the CLSI M45-A3 guideline was 5.4% (8/148). The incompatibility rates between the *E*-test and broth dilution methods for erythromycin, ciprofloxacin, and tetracycline were 8.0, 5.3, and 1.3%, respectively. The positive rates of the genes *cdtA* and *cdtC* were considerably higher in *Campylobacter jejuni* than in *C. coli*. Erythromycin is recommended as the first choice of treatment for campylobacteriosis. The disk diffusion method is suitable for prescreening *Campylobacter* susceptibility by using the CLSI M45-A2 and EUCAST criteria (low error rate of 3.4%). If antibiotic treatment fails or IZDs are between 6 and 20 mm, minimum inhibitory concentration testing by using the *E*-test method is highly recommended because the results of the *E*-test and broth dilution methods exhibit high agreement. The error rate of disk diffusion method using CLSI M45-A3 criteria is only slightly higher than B, which is also a suitable criteria.

## Introduction

*Campylobacter* spp. are zoonotic bacteria. First named in 1963, the *Campylobacter* genus currently comprises more than 20 species (Sebald and Veron, 1963). The main causes of human infection are *Campylobacter jejuni* and *Campylobacter coli* (van der Beek et al., 2010). There are a few reports of infections in other species (Man, 2011). People who are in close contact with animals, consume raw meat, are employed in animal husbandry, or own pets are at a high risk of campylobacteriosis (Dasti et al., 2010). *Campylobacter* infections may cause watery diarrhea, abdominal cramps or fever (Kaakoush et al., 2015). Severe campylobacteriosis has also been reported (Ge et al., 2013). Antibiotic treatment is suggested for patients with severe campylobacteriosis (Kaakoush et al., 2015). There are several common antimicrobial agents for erythromycin, ciprofloxacin, tetracycline, and doxycycline in campylobacteriosis therapy (Ge et al., 2013). The Clinical and Laboratory Standards Institute (CLSI) M45 guidelines recommend the susceptibility test by using the disk diffusion and broth dilution minimum inhibitory concentration (MIC) methods (Clinical and Laboratory Standards Institute, 2016). According to the M45-A2 (2010 version) guidelines, *Campylobacter* can be reported as being resistant to erythromycin and ciprofloxacin if no inhibition zone is observed in disk diffusion screening. If the inhibition zone diameter (IZD) is >6 mm, the susceptibility can be confirmed using the MIC method according to the interpretive criteria. The sensitivity of *Campylobacter* to tetracycline and doxycycline can only be confirmed using the broth micro-dilution method. In the M45-A3 (2016 version) guidelines, the IZD interpretive criteria were newly added, but the MIC interpretive criteria were the same as those in the previous version. However, the European Committee on Antimicrobial Susceptibility Testing (EUCAST) guidelines include another criterion for susceptibility, which is different from the CLSI criterion for susceptibility in *C. jejuni* and *C. coli* (EUCAST, 2018). Whether the use of the 2016 version criterion is feasible and whether the IZDs are consistent with the MIC results remain unclear in Taiwan. In addition, whether the results of the *E*-test method for MIC determination are consistent with those of the broth micro-dilution method, which is more complex and expensive than the *E*-test, requires investigation.

Studies have shown that the resistance of *Campylobacter* is increasing, particularly its resistance to commonly prescribed ciprofloxacin and tetracycline (Luangtongkum et al., 2009). Because most hospitals in Taiwan do not report the antimicrobial susceptibility of *Campylobacter* spp., the resistance of *Campylobacter* isolates is unknown in Taiwan. In this study, we intended to examine the antimicrobial susceptibility of *Campylobacter* to provide reliable data for clinical use and for reducing the dependence on empirical medication and literature recommendations when treating campylobacteriosis. In addition, susceptibility data from different years were analyzed to identify drug resistance trends. We analyzed susceptibility data from different areas in Taiwan to determine whether regional differences exist in antibiotic resistance.

*Campylobacter* produces several cytotoxins, including the extensively studied cytolethal distending toxin (CDT), which is also produced by many gram-negative bacteria (Bolton, 2015). *Campylobacter* species that produce toxins are more likely to cause serious illnesses than species that do not (Fais et al., 2016). Analysis of virulence factors is helpful to provide evidence for active treatment.

## Materials and Methods

### Analysis of Case Numbers of Campylobacteriosis

Analysis of the number of cases of campylobacteriosis in Chang Gung Memorial Hospital, Linkou, from 2005 to 2014 showed that the average number of cases of intestinal infection per year was 181. The average number of cases of parenteral infection per year was 9 in the same period; however, the number of cases was slightly higher in 2008–2011 probably because of the influence in the area (Supplementary Material A). The positive rate of virulence was higher in children than in adults, and the rate has not changed significantly from 2010 to 2014 (Supplementary Material B). No seasonal differences in the case numbers were observed, but the positive rate was higher in winter because fewer samples were collected in winter (Supplementary Material C). Intestinal infection was predominantly observed in children aged 1–6 years, accounting for 34.4% of the total case number; however, parenteral infection was predominantly observed in adults aged > 45 years, accounting for 68.6% of the total case number (Supplementary Material D).

### Bacterial Isolates and Growth Conditions

In total, 236 bacterial isolates were analyzed. The isolates consisted of 165 isolates from patients in northern Taiwan (2001–2012: 59 isolates of *C. jejuni* and 29 isolates of *C. coli*; 2013–2014: 45 isolates of *C. jejuni*, 31 isolates of *C. coli*, and 1 isolate of *C. fetus*) and 71 isolates from patients in southern Taiwan (2013–2014: 57 isolates of *C. jejuni* and 14 isolates of *C. coli*). Stool (193), blood (41), and dialysate (2) samples of the patients were collected. The clinical isolates obtained from northern and southern Taiwan were collected consecutively in different periods from two tertiary medical centers, namely Linkou Chang Gung Memorial Hospital and Kaohsiung Chang Gung Memorial Hospital, respectively. All samples were plated on Columbia blood agar plates containing 5% sheep blood agar and were incubated at 42°C in a microaerophilic atmosphere(CampyPak; BBL; Becton Dickinson, Rutherford, NJ, United States) for 24–48 h. Species identification was performed through matrix-assisted laser desorption ionization time of flight mass spectrometry and multiplex polymerase chain reaction (PCR) assay (Wang et al., 2002; Schulthess et al., 2013; Randall et al., 2015). The accuracy of MALDI-TOF mass identification in *Campylobacter* is close to 100% (Randall et al., 2015). The mass identifications and data analyses were performed using the Bruker LT microflex MALDI-TOF mass spectrometer (Bruker Daltonics, Bremen, Germany). A direct smear method with a 70% formic acid overlay was used. The gene of PCR is *hipO* from *C. jejuni*; glyA from *C. coli, C. lari*, and *C. upsaliensis*; *sapB2* from C. fetus subsp. fetus; and the internal control 23S rRNA.

### Broth Micro-Dilution Method

Minimum inhibitory concentration were determined for the 236 *Campylobacter* isolates using a commercial susceptibility plates (Sensititre, Trek Diagnostic Systems) containing serial double fold dilutions of nine antimicrobial agents, namely erythromycin, azithromycin, telithromycin, clindamycin, tetracycline, nalidixic acid, ciprofloxacin, florfenicol, and gentamicin. The broth dilution protocol was based on that provided in the CLSI guidelines (Clinical and Laboratory Standards Institute, 2016). The results of susceptibility to erythromycin, ciprofloxacin, and tetracycline were interpreted using epidemiological cutoff values based on MICs reported in the CLSI guidelines. The CLSI interpretive criteria for the other six antibiotics are not available. Quality control is the operation of *C. jejuni* ATCC 33560 specified by the manufacturer.

### Disk Diffusion Method

Susceptibility testing of erythromycin, tetracycline and ciprofloxacin was performed for 148 isolates of *Campylobacter* spp. obtained from patients in northern (*n* = 77) and southern (*n* = 71) Taiwan from 2013 to 2014 by using the disk diffusion method. The disk diffusion protocol, quality control and the interpretative criteria used were based on the CLSI guidelines (Clinical and Laboratory Standards Institute, 2016). Culture condition is Mueller Hintion agar with 5% sheep blood, 42°C for 48 h in a microaerophilic atmosphere(CampyPak; BBL; Becton Dickinson, Rutherford, NJ, United States).

### *E*-Test Method

The *E*-test was performed for 75 isolates (40 from northern and 35 from southern Taiwan) selected randomly from the 148 isolates obtained during 2013–2014; erythromycin, ciprofloxacin, and tetracycline were used for this test. The MICs of erythromycin, ciprofloxacin, and tetracycline for the *Campylobacter* isolates were determined using the standard *E*-test method. After inoculation with McFarland 0.5 turbidity standard *Campylobacter* culture, 90-mm plates containing *E*-test strips (AB BIODISK, Solna, Sweden) were incubated at 42°C for 48 h in a microaerobic environment (CampyPak; BBL; Becton Dickinson, Rutherford, NJ, United States). Quality control is the operation of *C. jejuni* ATCC 33560.

### Virulence Factor Analysis

The genes encoding CDT, namely *cdtA, cdtB, cdtC*, and *ceuE*, were detected in the 148 isolates obtained from 2013 to 2014 through PCR, as previously described (Bang et al., 2003). The primers sequence is *cdtA* (GNW: 5′-GGAAATTGGATTTGGGGCTATACT-3′; IVH: 5′-ATCACAAGGATAATGGACAAT-3′; Amp-licon: 165 bp), *cdtB* (VAT2: 5′-GTTAAAATCCCCTGCTATCAACCA-3′; WMI-R 5′-GTTGGCACTTGGAATTTGCAAGGC-3′; Amplicon: 495 bp), *cdtC* (WMI-F: 5′-TGGATGATAGCAGGGGATTTTAAC-3′; LPF-X: 5′-TTGCACATAACCAAAAGGAAG-3′; Amplicon: 555 bp), *ceuE* for *C. jejuni* (*ceuEJ*) (JEJ1: 5′-CCTGCTCGGTGAAAGTTTTG-3′; JEJ2: 5′-GATCTTTTTGTTTTGTGCTGC-3′; Amplicon: 794 bp), *ceuE* for *C. coli* (*ceuEC*) (COL1: 5′-ATGAAAAAATATTTAGTTTTTGCA-3′; COL2: 5′-ATTTTATTATTTGTAGCAGCG-3′;Amplicon: 894 bp). The conditions of PCR are 94°C1 min/42°C2 min/72°C3 min/(30 cycles) at *cdtA/cdtB/cdtC* and 95°C3 min/57°C30 s/72°C1min/30 cycles at *ceuEJ* and 95°C3 0 s/57°C30 s /72°C1 min/30 cycles at *ceuEC*.

### Statistical Analysis

The Student *t*-test was used to determine the significance of differences. A difference was considered statistically significant if *p* < 0.05.

### Ethics Approval and Consent to Participate

The isolate was not for the study, but for the treatment of infectious diseases in routine hospital laboratory procedure. We only used the bacterial isolate retained in the bacterium library, and the patient data are kept anonymous. Since this study only focuses on a bacterial isolate rather than patients, ethical approval was not necessary for the study according to the Swedish act concerning the ethical review of research involving humans, Etikprövningslagen (2003:460).

## Results

### Susceptibility Analysis

Antibiotic sensitivity was analyzed using the MIC and disk diffusion methods. Clinical isolates either from northern or southern Taiwan were used for analysis. The sensitivity rate for erythromycin detected using the MIC assay was 119/148 (80.4%) (Table 1A). Low drug resistance indicated that erythromycin is a suitable choice for empirical therapy. In the disk diffusion method, 24 (30.0%) isolates with small IZDs (≤6 mm) were all resistant, and 5 (4.0%) isolates with large IZDs (>6 mm) were resistant. Eight isolates exhibited different results from those reported in CLSI M45-A3 guidelines. Three isolates which diameter less than 13 mm were sensitive and five isolates larger than 16 mm were resistant. The high agreement between the two methods indicated that the disk diffusion method with erythromycin is useful for susceptibility screening. The resistant rates for ciprofloxacin and tetracycline determined through MIC assays were 138/148 (93.2%) and 143/148 (96.6%) (Tables 1B,C), respectively. High antimicrobial resistance showed that both these antibiotics are not suitable choices for empirical therapy. The isolates with small IZDs (≤6 mm) were all resistant to tetracycline, but not all isolates (129/132, 97.7%) were resistant to ciprofloxacin. The isolates with large IZDs (>6 mm) that had sensitivity rates of 11/16 and 15/20 were non-sensitive to ciprofloxacin and tetracycline, respectively. Unexpectedly, some of the isolates with very large IZDs (>40 mm) were found to be resistant in the MIC assay (Table 1). This disagreement between the results of the two methods indicates that the disk diffusion method with ciprofloxacin and tetracycline is not useful for susceptibility screening.

**Table 1A:** Compliance analysis between the inhibition zone diameters and MICs by using broth dilution method for erythromycin (A), ciprofloxacin (B), and tetracycline (C).

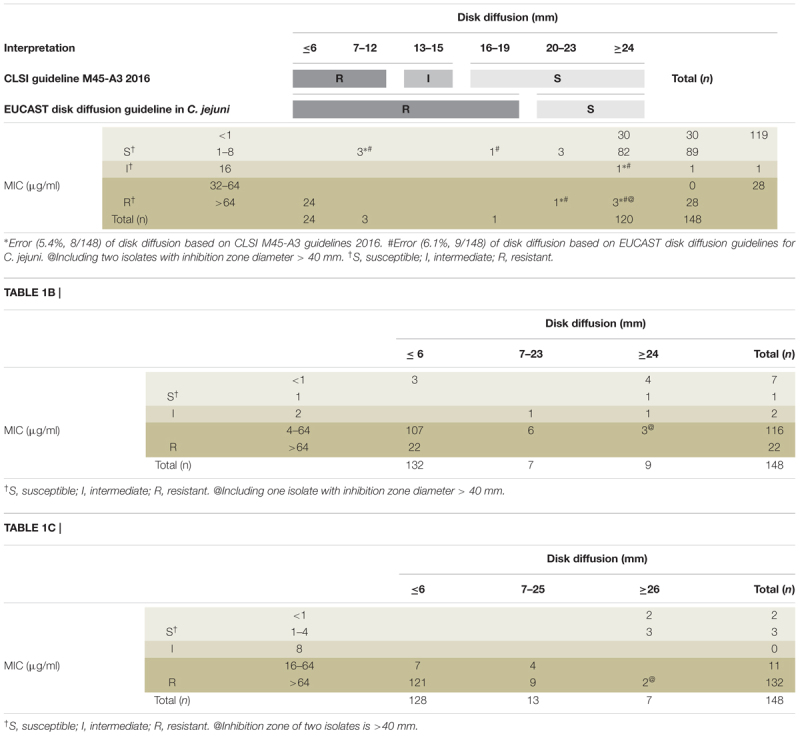

### Differences in Antibiotic Susceptibility Between the Isolates From Northern and Southern Taiwan

Several differences were observed in susceptibility to the nine antibiotics, namely erythromycin, azithromycin, telithromycin, clindamycin, tetracycline, nalidixic acid, ciprofloxacin, florfenicol, and gentamicin, between *Campylobacter* isolates from northern and southern Taiwan (Table 2). In *C. coli*, azithromycin resistance was higher in the isolates obtained from southern Taiwan than in those from northern Taiwan, but in *C. jejuni*, gentamycin resistance was higher in the isolates obtained from northern Taiwan than in those from southern Taiwan. Other antibiotic resistance rates (erythromycin, telithromycin, clindamycin, tetracycline, nalidixic acid, ciprofloxacin and florfenicol) were not significantly different between the isolates obtained from the two areas. Significant differences were observed in the antibiotic susceptibilities of *C. coli* and *C. jejuni* to erythromycin, azithromycin, telithromycin, clindamycin, and gentamicin in each area; however, no significant difference was observed between the isolates obtained from the two areas.

**Table 2:** Minimum inhibitory concentration operated by broth dilution of *C. jejuni* and *C. coli* isolated from patients in two tertiary medical centers in northern and southern Taiwan.

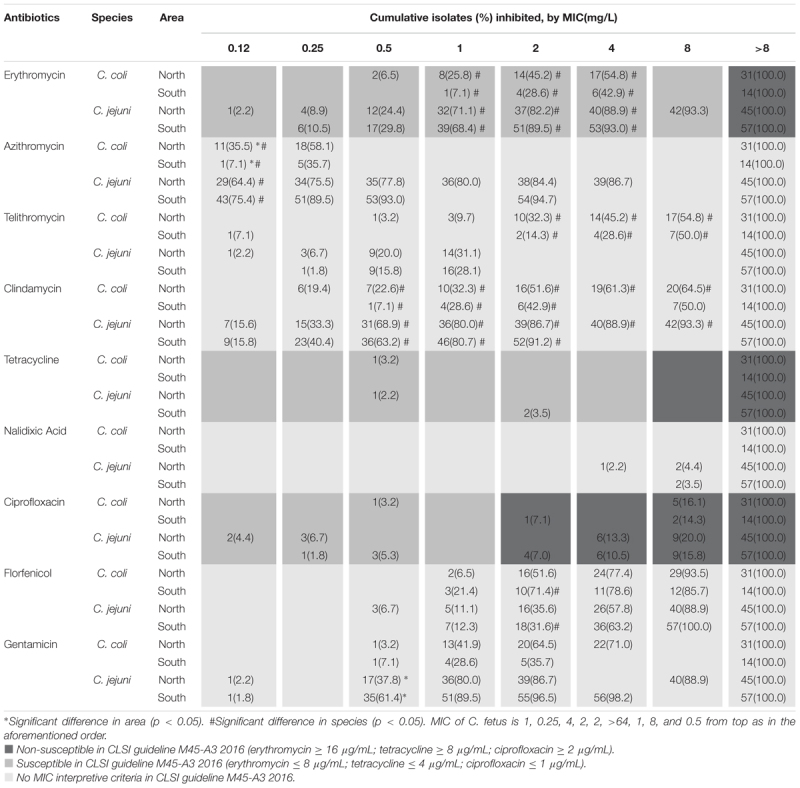

### Verification Analysis by Using the *E*-Test and Microdilution MIC Methods

The MICs of three antibiotics, namely erythromycin, ciprofloxacin, and tetracycline, in 75 *Campylobacter* isolates were further verified using the *E*-test and microdilution methods. As shown in Table 3, several disagreements were observed between the results of the two methods. The incompatibility rates for erythromycin, ciprofloxacin, and tetracycline were 8.0% (6/75), 5.3% (4/75), and 1.3% (1/75), respectively. Furthermore, the major error rates of erythromycin and ciprofloxacin are 6.7% (5/75) and 5.3% (4/75), and tetracycline has no major errors.

**Table 3:** Comparison between the *E*-test and broth-dilution methods for erythromycin **(A)**, ciprofloxacin **(B)**, and tetracycline **(C)**.

	Antibiotics		*E*-test		
			
			Susceptible	Intermediate	Resistant
Broth-dilution	**(A) Erythromycin**	Susceptible	59	1^∗^	3^∗#^
		Intermediate	0	0	0
		Resistant	2^∗#^	0	10
					
	**(B) Ciprofloxacin**	Susceptible	3	0	1^∗#^
		Intermediate	0	0	0
		Resistant	3^∗#^	0	68
					
	**(C) Tetracycline**	Susceptible	3	0	0
		Intermediate	0	0	0
		Resistant	0	1^∗^	71


*^∗^Inconsistency between different methods. #Major error. Interpretive criteria is based on the CLSI M45-A3 guidelines, 2016. Erythromycin: ≤8, 16, and ≥32 μg/mL; ciprofloxacin: ≤1, 2, and ≥4 μg/mL; tetracycline: ≤4, 8, or ≥16 μg/mL as susceptible, intermediate, or resistant.*

### Presence of Bacterial Virulence Genes

The genes encoding CDT, namely *cdtA, cdtB, cdtC, ceuEJ*, and *ceuEC*, were analyzed in the related *Campylobacter* species *C. coli* and *C. jejuni* (Table 4). The positive rates of *C. jejuni* for *cdtA* and *cdtC* in stool are 97.9% and 62.7%, respectively, and those for *C. coli* is 21.9% and 15.4% respectively. The positive rates for *cdtA* and *cdtC* were considerably higher in *C. jejuni* than in *C. coli* (*p* < 0.05). However, the positive rates of the other three virulence factors were nearly 100%, and no differences were observed between *C. coli* and *C. jejuni*.

**Table 4:** Positive rate of the presence of each *cdt* gene in clinical isolates.

*Campylobacter* isolate (n)	Specimen (n)	*cdtA*	*cdtB*	*cdtC*	*ceuEJ*	*ceuEC*
*C. coli* (45)	Other (6)^∗^	0.0%	100.0%	0.0%	N/A	100.0%
	Stool (39)	21.9%	100.0%	15.4%	N/A	100.0%
*C. fetus* (1)	Other (1)^∗^	0.0%	100.0%	100.0%	N/A	N/A
*C. jejuni* (102)	Other (6)^∗^	83.3%	100.0%	83.3%	100.0%	N/A
	Stool (96)	97.9%	100.0%	62.7%	99.0%	N/A


*^∗^The other specimens are blood (*n* = 11) and dialysate (*n* = 2). N/A, not applicable.*

### Antibiotic Susceptibility Analysis of *Campylobacter* Species Isolated From Patients in Different Periods

We further analyzed antibiotic resistance rates in three periods, namely 2001–2006 (*n* = 24), 2007–2012 (*n* = 64), and 2013–2014 (*n* = 77) in northern Taiwan (Table 5). The resistance rates for all antibiotics, except tetracycline, were high in *Campylobacter* isolated from blood than those from stool samples. Although the resistance rate for tetracycline in 2007–2012 was slightly low and the resistance rate for erythromycin in blood isolates was slightly high; the difference was not statistically significant.

**Table 5:** Antibiotic resistance rates of *Campylobacter* from 2001 to 2014 in northern Taiwan.

	2001–2006			2007–2012			2013–2014		
			
	Stool(16) %	Blood(8) %	Total(24) %	Stool(42) %	Blood(22) %	Total(64) %	Stool(67) %	Blood(10) %	Total(77) %
Ciprofloxacin	75.0	100.0	83.3	76.2	86.4	79.7	76.1	80.0	76.6
Erythromycin	18.8	25.0	20.8	14.3	18.2	15.6	19.4	40.0	22.1
Tetracycline	81.3	100.0	87.5	69.0	77.3	71.9	97.0	90.0	96.6


## Discussion

The antibiotic resistance interpretive criteria in the CLSI M45-A2 guidelines suggest that erythromycin is resistant when an IZD (≤6 mm) is not observed in a 15-μg paper disk, and uncertain if the IZD exceeds 6 mm. When the resistance results of the disk diffusion methods are uncertain, the MIC method can be used to determine drug resistance; accordingly, an MIC of ≥32 μg/mL represents that the isolate is resistant to the tested drug, whereas MICs of ≤8 and 16 μg/mL represent that the isolate is sensitive and intermediate, respectively. In addition to CLSI, other reference standards, such as EUCAST, are used in Europe. EUCAST also recommends the use of a 15-μg paper disk of erythromycin to test the susceptibility of *C. jejuni*. According to the criteria for *C. jejuni*, IZD < 20 mm indicates resistance, whereas IZD ≥ 20 mm indicates sensitivity; however, for *C. coli*, IZD < 24 mm indicates resistance, whereas IZD ≥ 24 mm indicates sensitivity. In addition, according to the criteria of the MIC method in EUCAST, an MIC of >4 μg/mL indicates resistance, whereas an MIC of ≤4 μg/mL indicates sensitivity, without an intermediate category. In the CLSI M45-A3 guidelines, disk diffusion criteria were added (IZD ≤ 12 mm indicates resistance, an IZD of 13–15 mm indicates the intermediate category, and IZD ≥ 16 mm indicates sensitivity).

Currently, antibiotic susceptibility testing for *Campylobacter* is not widely implemented in Taiwan. However, based on the needs of clinical treatment, susceptibility testing may be introduced in the future (Ge et al., 2013). Our hospital, similar to other hospitals in Taiwan, uses the antibiotic resistance interpretive criteria of the CLSI guidelines. However, we wish to identify the difference between these two criteria and between different antibiotic susceptibility testing methods.

In this study, all the 24 isolates with IZDs ≤ 6 mm for erythromycin showed MIC > 16 μg/mL (CLSI criteria). However, 4 of the 28 isolates considered resistant according to the EUCAST criteria (IZD < 20 mm) were sensitive according to the MIC method and CLSI criteria (MIC ≤ 8 μg/mL). The isolates with IZD ≥ 20 mm were considered sensitive according to the EUCAST criteria. Most of the isolates were sensitive, with the exception of five isolates with MIC > 8 μg/mL, which denotes the resistance criterion of CLSI. The discrepancies between disk diffusion and IZD < 20 mm (sensitive) and IZD ≥ 20 mm (resistant) are 14.2% (4/28) and 4.1% (5/120), respectively. We believe that the criteria of the disk diffusion method in EUCAST or CLSI for determining erythromycin susceptibility are feasible, despite a few errors, because the requirements of labor, material, resources, and turn-around-time for the disk diffusion method are less than those for the MIC method. Al-Natour’s study on *Campylobacter* isolated from animals showed a high degree of agreement between the two methods although the criteria used were different from those used in the present study (Al-Natour et al., 2016). However, our study showed that the disk method was only suitable for erythromycin and not for ciprofloxacin and tetracycline. The difference in suitability may be due to differences in drug resistance in isolates from different regions.

Although the erythromycin-resistant isolates identified using the M45-A2 criteria (IZD < 6 mm represents resistance) of CLSI were entirely correct, the use of these criteria is complicated for the isolates for which the resistance cannot be determined because of the discrepancy of intermediate isolates in the M45-A3 criteria and the complexity of the broth dilution MIC method. The combination of the CLSI and EUCAST criteria might provide additional information, such as ≤6 mm represents resistance, where >20 mm represents sensitivity (low error rate of 3.5%, 5/144). Because of a high error rate (75%, 3/4), if the IZD is between 6 and 20 mm or if antibiotic treatment failed, the MIC method is recommended.

Among the isolates, resistance to ciprofloxacin and tetracycline, regardless of the CLSI or EUCAST criteria, was high (>90%), thus indicating that these two antibiotics are not suitable for empirical treatment. Even if the IZD exceeded the “sensitive” criteria of EUCAST (≥26 mm for ciprofloxacin and ≥30 mm for tetracycline), a large proportion of the isolates were shown to be resistant according to the results of the MIC method. For ciprofloxacin, the rate of non-compliance was 33.3% (3/9) based on the CLSI MIC criteria and >56% based on the EUCAST MIC criteria. For tetracycline, the rate of non-compliance was 50% on average. The sensitivity to these two antibiotics could not be determined using the disk diffusion method; hence, the MIC method was used. Two methods of MIC determination are routinely used, broth dilution and *E*-test. The broth dilution method requires the preparation of antibiotic solutions or commercial kits that are expensive (US$25/isolate and nine antibiotics) and require more complicated operations. Commercial kits have a short shelf life, and are associated with difficulty of interpretation; however, the *E*-test is considerably simpler and have a long shelf life. Although the price of *E*-test for nine antibiotics is similar to that of broth dilution, the price of *E*-test are more flexible in price because we can choose a few antibiotics we need. We compared the rates of compliance of the two MIC methods to determine whether we can use the *E*-test result instead of the broth dilution method to obtain MIC data. The results showed that *E*-test method results exhibit high agreement with those of the broth dilution method according to the CLSI criteria, 94.6% (71/75), 94.6% (71/75), and 98.6% (74/75) for erythromycin, ciprofloxacin, and tetracycline, respectively.

Although previous studies have indicated an increase in *Campylobacter* resistance in recent years (Luangtongkum et al., 2009), no significant difference was obtained among the data of different years in our analysis, thus suggesting that the increase in ciprofloxacin and tetracycline resistance may have occurred before 2001. According to a study in Taiwan duck farms from 2013 to 2014, antimicrobial resistance differs between northern and southern Taiwan (Department of Veterinary Medicine, National Taiwan University, unpublished data). This discrepancy may be due to differences in the use of antibiotics in the livestock sector or in the natural distribution of such zoonotic bacteria. Because human infection partly results from meat consumption, we analyzed the regional difference of antibiotic susceptibility in patients with campylobacteriosis. We analyzed the differences in resistance to nine antibiotics between two tertiary medical centers, Linkou and Kaohsiung Chang Gung Memorial Hospitals, with a distance of 250 km between them, which represent northern and southern Taiwan, respectively. The results showed that the effects of regional differences on human infection were not significant because we observed differences in only two analysis groups, namely gentamycin for *C. jejuni* and azithromycin for *C. coli*, with higher and lower resistance, respectively, in the northern than in the southern area.

The number of *Campylobacter* bacteremia cases was highest in the middle-aged population in our study; the result is similar to that of a recent study in Israel in 2016 (Hussein et al., 2016). In our study, however, we did not find *Campylobacter* species other than C. *jejuni, C. coli*, and *C. fetus*, although the increasing prevalence of uncommon *Campylobacter* has been reported in the literature. Differences between species were observed in four antibiotics, namely azithromycin, erythromycin, telithromycin and clindamycin; *C. coli* showed higher resistance to all the drugs than *C. jejuni* did. Pigs are the main natural reservoirs of *C. coli*. We do not know whether the discrepancy is due to differences in the feeding process of pigs between regions or livestock or other differences such as the nature of species. According to a report by Riley in Canada, *C. coli* is more resistant to ciprofloxacin and erythromycin than *C. jejuni* (Riley et al., 2015). Moreover, our study reported that *C. coli* was less resistant to tetracycline than other species. Similar studies have provided different results on drug resistance (El-Adawy et al., 2015; Cha et al., 2016; Kim et al., 2016). Therefore, the establishment of a bacterial local susceptibility database is required because of the regional difference.

*Campylobacter* is more toxic to hosts when it contains virulence factors (Bolton, 2015; Lai et al., 2016). We analyzed the differences between four virulence factor genes, namely *cdtA, cdtB, cdtC*, and *ceuE*, between *C. jejuni* and *C. coli*. The results showed that the positive rate of *cdtA* and *cdtC* in *C. jejuni* (*cdtA*: 97.9% in stool and 83.3% in the other; *cdtC*: 62.7% in stool and 83.3% in the other) was significantly higher than that in *C. coli* (*cdtA*: 21.9% in stool and 0.0% in the other; *cdtC*: 15.4% in stool and 0.0% in the other). However, the difference of positive rate in *cdtB* and *ceuE* between *C. jejuni* and *C. coli* is not significant due to their positive rate nearly 100%. According to a study of *Campylobacter* in storks in Europe (Szczepanska et al., 2015), the positive rate of *cdtB* in *Campylobacter* was 58–88%, which is less than that observed in our study. The positive rate of *C. coli* in the above study was higher than that of *C. jejuni*, which was also different from our human clinical case study. We believe that this difference is attributable not only to differences in regions but also differences in species. However, the number of *Campylobacter* studies on the positive rate of *cdt* genes in human clinical infection cases has not increased in recent years. Although the positive rate of the virulence factor in *C. jejuni* is higher than in *C. coli*, the number of parenteral infection cases (*C. jejuni*: *C. coli* = 1:1) caused by *C. coli* is higher than that of intestinal infection cases (*C. jejuni*: *C. coli* = 1:0.4). The use of antibiotics and other invasive factors may contribute to these differences. Additional studies are needed to explain these differences.

## Conclusion

Cases of severe or invasive campylobacteriosis require the use of antibiotics; erythromycin is suitable as the first choice, and the ciprofloxacin and tetracycline are not suitable because of their high drug resistance (observed in more than 95% of cases in Taiwan). The disk diffusion method with erythromycin can be used as a susceptibility screening method, as determined using the CLSI (≤6 mm represents resistance) and EUCAST (≥20 mm represents sensitivity) criteria. The error rate of the combination method was only 3.5% (5/144). If antibiotic treatment fails or when the IZD is between 6 and 20 mm, the MIC method is recommended. The error rate of disk diffusion method using CLSI M45-A3 criteria is only slightly higher than B, which is also a suitable criteria. The *E*-test is an alternative approach for the broth dilution method because of the high agreement of its results with broth dilution results.

## Data Availability Statement

The raw data supporting the conclusions of this manuscript will be made available by the authors, without undue reservation, to any qualified researcher.

## Author Contributions

M-CG, S-FK, and J-JL designed the experiments. C-CC, and H-LY integrated the experiments for bacterial isolates and control of antibiotic susceptibility test. M-CG and S-FK participated in other experiments. S-CC performed the statistical analyses. M-CG and J-JL wrote the manuscript. All authors discussed the results and implications and commented on the manuscript at all stages.

## Conflict of Interest Statement

The authors declare that the research was conducted in the absence of any commercial or financial relationships that could be construed as a potential conflict of interest.
