# Supplementary material for: Antimicrobial Susceptibility and Virulence Surveillance of Campylobacter spp. Isolated From Patients in Two Tertiary Medical Centers in Taiwan
Source: Front Microbiol. 2019 Jan 7;9:3186. doi: 10.3389/fmicb.2018.03186 (PMC6330319; doi:10.3389/fmicb.2018.03186)
Supplement: Supplementary file 1 [file Table_1.DOCX]

Supplementary Material

Antimicrobial Susceptibility and Virulence Surveillance of *Campylobacter* spp. Isolated from Patients in two Tertiary Medical Centers in Taiwan

Mao-Cheng Ge, Shu-Fang Kuo, Shih-Cheng Chang, Chun-Chih Chien, Huey-Ling You and Jang-Jih Lu*

***Correspondence:** Jang-Jih Lu: [jjlpcp@adm.cgmh.org.tw](mailto:jjlpcp@adm.cgmh.org.tw)

1. Case numbers of intestinal and parenteral *Campylobacter* infection from 2005 to 2014 in Linkou Chang Gung Memorial Hospital

| **year** | **2005** | **2006** | **2007** | **2008** | **2009** | **2010** | **2011** | **2012** | **2013** | **2014** |
| --- | --- | --- | --- | --- | --- | --- | --- | --- | --- | --- |
| **intestinal cases (no.)** | **144** | **181** | **183** | **228** | **203** | **221** | **176** | **168** | **156** | **154** |
| **parenteral case (no.)** | **6** | **4** | **5** | **14** | **11** | **14** | **12** | **6** | **8** | **11** |

B. The positive rates of the *Campylobacter* in adults (>18) and children (≤18) from 2010 to 2014 in Linkou Chang Gung Memorial Hospital

|  |  |  |  |  |  |  |
| --- | --- | --- | --- | --- | --- | --- |
| **year** | **2010** | **2011** | **2012** | **2013** | **2014** | **total** |
| **speciments (no.)** | **4412** | **4039** | **4092** | **4391** | **4282** | **21216** |
| **positive cases (no.)** | **235** | **188** | **169** | **159** | **200** | **951** |
| **positive rate (%)** | **5.3** | **4.7** | **4.1** | **3.6** | **4.7** | **4.5** |
|  |  |  |  |  |  |  |
| **speciments in adults (no.)** | **1220** | **1250** | **1200** | **1117** | **1260** | **6047** |
| **positive cases in adults (no.)** | **39** | **36** | **38** | **29** | **39** | **181** |
| **positive rate in adults (%)** | **3.2** | **2.9** | **3.2** | **2.6** | **3.1** | **3.0** |
|  |  |  |  |  |  |  |
| **speciments in children (no.)** | **3192** | **2789** | **2892** | **3274** | **3022** | **15169** |
| **positive cases in children (no.)** | **196** | **152** | **131** | **130** | **161** | **770** |
| **positive rate in children (%)** | **6.1** | **5.1** | **4.5** | **4.0** | **5.3** | **5.1** |

C. Average positive rates in different months from 2005 to 2014 in Linkou Chang Gung Memorial Hospital

|  |  |  |  |  |  |  |  |  |  |  |  |  |
| --- | --- | --- | --- | --- | --- | --- | --- | --- | --- | --- | --- | --- |
| **month** | **1** | **2** | **3** | **4** | **5** | **6** | **7** | **8** | **9** | **10** | **11** | **12** |
| **stool speciments (no.)** | **1539** | **1412** | **1779** | **1700** | **1916** | **1883** | **2015** | **2135** | **1902** | **1720** | **1612** | **1603** |
| **positive cases (no.)** | **81** | **72** | **80** | **76** | **70** | **79** | **86** | **78** | **77** | **77** | **76** | **99** |
| **positive rate (%)** | **5.3** | **5.1** | **4.5** | **4.5** | **3.7** | **4.2** | **4.3** | **3.7** | **4.0** | **4.5** | **4.7** | **6.2** |

D. Distribution of intestinal and parenteral infection cases in Linkou Chang Gung Memorial Hospital

| **age** | **≦1** | **1~≦6** | **6~≦12** | **12~≦18** | **18~≦45** | **45~≦60** | **>60** |
| --- | --- | --- | --- | --- | --- | --- | --- |
| **intestinal cases (no.)** | **7.0% (61)** | **34.4% (301)** | **21.7% (190)** | **12.5% (109)** | **12.8% (112)** | **5.0% (44)** | **6.6% (58)** |
| **parenteral cases (no.)** | **3.9% (2)** | **2.0% (1)** | **2.0% (1)** | **0.0% (0)** | **23.5% (12)** | **43.1% (22)** | **25.5% (13)** |
